# Unequivocal identification of intracellular aluminium adjuvant in a monocytic THP-1 cell line

**DOI:** 10.1038/srep06287

**Published:** 2014-09-05

**Authors:** Matthew Mold, Håkan Eriksson, Peter Siesjö, Anna Darabi, Emma Shardlow, Christopher Exley

**Affiliations:** 1 The Birchall Centre, Lennard-Jones Laboratories, Keele University, Keele, Staffordshire, ST5 5BG, UK; 2 Department of Biomedical Laboratory Science, Faculty of Health and Society, Malmö University, SE-205 06 Malmö, Sweden; 3 Glioma Immunotherapy Group, Department of Clinical Sciences, BMC D14, Lund University, SE-221 84 Lund, Sweden

## Abstract

Aluminium-based adjuvants (ABA) are the predominant adjuvants used in human vaccinations. While a consensus is yet to be reached on the aetiology of the biological activities of ABA several studies have identified shape, crystallinity and size as critical factors affecting their adjuvanticity. In spite of recent advances, the fate of ABA following their administration remains unclear. Few if any studies have demonstrated the unequivocal presence of intracellular ABA. Herein we demonstrate for the first time the unequivocal identification of ABA within a monocytic T helper 1 (THP-1) cell line, using lumogallion as a fluorescent molecular probe for aluminium. Use of these new methods revealed that particulate ABA was only found in the cell cytoplasm. Transmission electron microscopy revealed that ABA were contained within vesicle-like structures of approximately 0.5–1 μm in diameter.

Since their inception in the 1920s aluminium based adjuvants (ABA) have remained the predominantly used adjuvants in human vaccinations^1^. ABA are aluminium salts dispersed in water to form heterogeneous suspensions or gels, of hydrated colloid particles that consist of micron-sized aggregates of 1–20 μm with primary particles in the nano-size range^2^,^3^,^4^. Aluminium oxyhydroxide (AlO(OH)) adjuvants such as the licensed ABA Alhydrogel® (Brenntag Biosector, Denmark)^5^ are the most commonly used manufactured ABA in clinical vaccinations. AlO(OH)-based adjuvants are principally used owing to being the most well-defined and consistent of the clinically approved ABA and in their ability to adsorb negatively charged protein antigens from aqueous solutions at physiological pH^2^.

When formulated in vaccine preparations, ABA both potentiate the efficacy of weak antigens and shape the resultant immune response^6^. Use of antigen only often results in weak immunopotentiation and little or no antibody production^7^. Whilst the efficacy of ABA is undisputed, a consensus is yet to be reached upon their biological activities *in vivo*. ABA including aluminium hydroxide preparations are used in vaccinating against diphtheria, hepatitis A and B and against human papilloma virus (HPV) to prevent cervical cancers^6^,^7^. An ever intensifying research effort is currently underway to explain the observed adjuvanticity of ABA with several studies highlighting their physicochemical properties as a key determinant. Interestingly it has been shown that nanoparticles of aluminium hydroxide of *ca* 200 nm elicit more potent adjuvant activities in an *in vivo* model of murine adjuvanticity, than larger particles in the micron-size range^9^. The increased adjuvanticity of aluminium hydroxide nanoparticles was explained by their greater ease of uptake by dendritic antigen presenting cells (APCs)^9^.

Variation of the physicochemical properties of AlO(OH)-based adjuvants via engineering particles with defined shape, size and morphology characteristics has been used in studying their immunopotentiating properties^10^. Recent research by Sun and co-workers (2013) showed that the design of ordered rod-like AlO(OH) nanoparticles with higher crystallinity resulted in greater cellular uptake of the antigen and increased IL-1β production^10^. Interestingly, increased production of IL-12 and IL-6 was observed in bone marrow-derived dendritic cells (BMDCs) for the engineered AlO(OH) nanorods, the former being a known polariser of a Th1 response^7^. Therefore alteration of the physicochemical properties of aluminium hydroxide based adjuvants alone has been shown to shape their immunostimulatory properties both *in vivo* and *in vitro*^9^,^10^.

Surprisingly, little experimental information has eluded to the potential cellular uptake of ABA or any compartmentalisation of ABA, inside the cell^3^,^11^,^12^,^13^,^14^,^15^. This has been suggested to be critical for the activation and assembly of the Nalp3 inflammasome, driving pro-inflammatory immune responses via secretion of mature interleukin IL-1β and IL-18 cleaved from the prointerleukins by actvated caspase-1^16^,^17^. Ultrastructural investigations paired with X-ray microanalysis have previously been used to successfully confirm the presence of aluminium in the skin^13^.

Aluminium hydroxide granulomas from excised subcutaneous nodules were identified in three children ranging in age from 18 to 36 months following injection of a diphtheria, tetanus and pertussis vaccine, adjuvanted with aluminium hydroxide^13^. Whilst aluminium was identified in the skin the aluminium signal measured was not confined to any intracellular environment and hence evidence of intracellular aluminium was neither inferred by the authors nor could it be established unequivocally^13^.

Further X-ray based methods have used particle-induced X-ray emission (PIXE) to identify aluminium in quadriceps muscle of Cynomolgus monkeys, following intramuscular administration of a Diphtheria-tetanus vaccine containing 0.6 mg/mL of either AlO(OH) or aluminium hydroxyphosphate (AlPO_4_) based adjuvants^14^. Increases in the aluminium concentration were found at a muscular level over scanned areas of 1 mm^2^ in the injection site, with marked increases identified in muscular lesions following 3 and 6 months post-injection^14^. No results were included however to show that aluminium was localised within cells.

A study by Rimaniol and co-workers (2004) demonstrated the presence of crystalline inclusions in macrophages exposed to 2 μg/mL aluminium oxyhydroxide (AlO(OH)) adjuvant via transmission electron microscopy (TEM)^15^. Electron micrographs revealed electron dense material scattered throughout macrophages. In spite of the observations of electron dense material in macrophages, no direct evidence was provided to confirm that those inclusions identified contained particulate AlO(OH)^15^. A more recent study by Lu & HogenEsch (2013) highlighted the presence of an ABA (Rehydragel™ HPA, USA) in murine macrophages using morin (2,3,4,5,7-pentahydroxy- flavone) staining^11^.

An earlier investigation using the same method found ABA internalised in epithelioid macrophages and multinucleated giant cells in rhesus macaque skin sections^12^. No autofluorescence controls were shown in either paper however and the influences of the protein antigens alone were not addressed^11^,^12^. Morin is also known to bind to magnesium (Mg^2+^) and calcium (Ca^2+^) ions to produce false positives^18^. A hydrochloric acid pre-rinse, prior to staining is frequently used to account for such. The resultant acidity however, is likely to promote the formation of soluble Al^3+^ increasing the likelihood of the dissolution of aluminium bound in tissue and its possible migration throughout the tissue^19^.

Investigations monitoring the potential cellular uptake of ABA have preferentially labelled the adsorbed protein antigen (often ovalbumin (OVA)) with the amine-reactive derivative of the fluorescein dye, fluorescein isothiocyanate (FITC)^9^,^10^,^20^,^21^. Following adsorption of an ABA with FITC-labelled OVA, the presence of the adjuvant has been inferred intracellularly in cultured DC-lines by use of confocal microscopy^20^,^21^, fluorescence microscopy^9^ and flow cytometry^10^,^20^,^21^. Whilst the presence of the OVA protein antigen has been observed intracellularly within APCs^9^,^20^,^21^, whether the adsorbed ABA is internalised or not remains to be determined as the use of FITC only allows for the identification of internalised antigen.

Pre-labelling AlO(OH) adjuvants with FITC via the use of pre-incubation with organic moieties^10^ may also result in changes to the surface of the particulates as well as modifications to their hydroxyl functionality and size. This may in turn affect their uptake as the variation of such physicochemical properties of ABA has been shown to dramatically affect their cellular uptake^8^,^9^,^10^. Furthermore, potential modifications to the endocytic/phagocytic pathways of internalisation following conjugation of the ABA to FITC-OVA cannot be ruled out.

Use of AlO(OH) (Alhydrogel, Brenntag) formulated with the chimaeric Ealpha green fluorescent protein (EαGFP) demonstrated an increase in antigen uptake compared to uptake of EαGFP alone via the detection of GFP using flow cytometry^22^. As with the use of FITC only the presence of the antigen could be inferred inside the cell of which the authors summarised that ABA provide an enhanced delivery mechanism of the antigen only whilst not addressing potential uptake of the adjuvant by dendritic cells (DCs)^22^. Additional studies have supported these conclusions with crystals of the ABA being suggested to not enter the cell and rather aid in the transport of the antigen across DC membranes with activation of the Nalp3 inflammasome being dispensable for triggering subsequent immune responses^23^.

Few if any studies within the literature have therefore shown the direct monitoring of the intracellular ABA content of various immune cells and a consensus upon the biological activates of ABA has yet to be reached^16^,^17^. Herein we report for the first time the unequivocal identification of intracellular AlO(OH) particles both for a clinically approved and an experimental AlO(OH)-based adjuvant in a monocytic T-helper 1 (THP-1) cell line. Positive identification of particulate ABA was observed intracellularly by use of the planar tridentate ligand lumogallion [4-chloro-3-(2,4-dihydroxyphenylazo)-2-hydroxybenzene-1-sulphonic acid] as a fluorescent molecular probe for aluminium^24^.

Lumogallion is commonly used to detect the presence of aluminium at low concentrations in natural waters with a limit of detection of *ca* 2 nM^25^ and has been used extensively for the identification of the metal ion in plant roots^26^,^27^. Lumogallion has been suggested to act as a planar tridentate ligand for aluminium with binding of the metal ion via the two phenolic oxygen ions and the azo (N = N) group of lumogallion establishing a 1:1 stoichiometry of the complex^24^. Lumogallion is known to bind to the soluble ionic Al^3+^ form of aluminium, with complex formation resulting in an emission intensity at *ca* 590 nm (orange fluorescence) upon an excitation wavelength of *ca* 500 nm^28^.

Unlike morin, lumogallion is not known to be prone to false positives including metal ions commonly found in physiological media including magnesium, calcium^24^ and iron^25^. In combination with fluorescence microscopy, use of the complementary techniques, confocal microscopy and TEM further aided to confirm the intracellular presence of particulate aluminium at a physiologically relevant pH, furthering our understanding into the role of ABA in clinical vaccinations.

## Methods

### Cell culture and fixation

All chemicals were from Sigma Aldrich, UK, unless otherwise stated. THP-1 cells (ATCC TIB-202, LGC Standards, UK) were cultured in complete R10 medium which consisted of RPMI 1640 medium supplemented with 10% foetal calf serum (EU grade) and 100 μg/mL gentamicin (both from PAA laboratories, Linz, Austria). All cells were cultured at 37°C in a humidified atmosphere containing 5% CO_2_.

Native THP-1 cells (i.e. those containing no ABA) were cultured in the presence of complete R10 medium only, whilst those cell treatments containing the ABA were co-cultured in R10 media containing 50 or 200 μg/mL of either the aluminium oxyhydroxide-based (AlO(OH)) adjuvant, AlO(OH)^Sigma^ (A8222, aluminium hydroxide gel colloidal suspension, Sigma Aldrich, UK) or Brenntag's Alhydrogel® (2%), AlO(OH)^Brenntag^ (Brenntag Biosector, Denmark) for 24 h. Each of the cell treatments were cultured in triplicate in 96 well plates with a final volume of 200 μL per well. Following incubation the triplicates made from each of the co-culture conditions were pooled and the cells were subsequently centrifuged and washed with phosphate buffered saline (PBS).

Cells from each of the treatments were then fixed by transfer into 1% paraformaldehyde (PFA) and incubating for 24 h, at ambient temperature. The number of intact cells after fixation was counted using an Accuri C6 flow cytometer. After counting, each of the cell treatments were re-suspended in 70% *v/v* ethanol in order to prevent over fixation of the cells resulting in the reduced penetration of histochemical reagents.

### Paraffin and resin embedding of THP-1 cells

Full details concerning paraffin and resin embedding of THP-1 cells for sectioning are detailed in the supplementary methods. Briefly, PFA fixed THP-1 cells co-cultured in R10 medium in the absence or presence of AlO(OH)^Sigma^ were pre-embedded into agar and dehydrated fully through an ethanol gradient. For paraffin embedding the resultant agar-cell blocks were cleared with Histo-Clear and infiltrated with paraffin wax. 2 μm sections were prepared by use of a rotary microtome and the agar-cell sections were rehydrated back into ultrapure water prior to staining. THP-1 cells for resin embedding were dehydrated as above and then gradually infiltrated with Spurr resin. Polymerised resin blocks were sectioned at 100 nm by use of an automated ultramicrotome.

### Cryosectioning

Fixed THP-1 cells co-cultured in complete R10 medium in the presence or absence of 50 or 200 μg/mL AlO(OH)^Brenntag^ adjuvant (24 h) were pelleted and mixed with 40 μl Tissue-Tek® O.C.T compound, Sakura® Finetek cryoprotection medium (VWR, Stockholm, Sweden). Cells were then transferred to a 10 × 10 × 5 mm Tissue-Tek® Cryomold® (HistoLab®, Gothenburg, Sweden) and frozen on dry ice. The frozen cells were sectioned at 6 μm using a Leica CM 3050 cryostat and mounted on Menzel SuperFrost® Plus glass slides (VWR, Stockholm, Sweden). Slides were stored at −80°C until further processing.

### Lumogallion staining of cell sections

Agar-cell sections were de-waxed and rehydrated by transferring the slides in a holder (up to 12 at a time) through 250.0 mL of the following in glass staining dishes: Histo-Clear for 3 min, fresh Histo-Clear for 1 min, 100% ethanol for 2 min, 95, 70, 50 & 30% *v/v* ethanol for 1 min and finally ultrapure water for 35 s. Sides were agitated in each solution and blotted dry on tissue paper between each stage. Rehydrated cell sections were immediately placed into either 100 μM lumogallion (TCI Europe N.V. Belgium) buffered in 50 mM PIPES, pH 7.4 or the PIPES-buffer alone for auto-fluorescence analyses and were then covered and incubated at ambient temperature in the dark for 24 h.

Following 24 h the slides were removed, blotted dry and for the lumogallion stained sections only, were rinsed by gentle agitation in 50 mM PIPES, pH 7.4 for 2 min. Slides were rinsed in ultrapure water for 2 min, air dried and finally mounted using ProLong® Gold Antifade Reagent with 4′,6-diamidino-2-phenylindole, dihydrochloride (DAPI) (Life Technologies, UK). Slides were stored horizontally for 24 h at 4°C to allow the mounting media to fully cure, prior to their analysis via fluorescence microscopy. Cryosectioned THP-1 cells were thawed for 10 min at ambient temperature and then placed into either PIPES-buffer alone or PIPES-buffered lumogallion. Cryosectioned THP-1 cells were stained and mounted in the exact same way as the agar-cell sections.

### Fluorescence microscopy

For specific details pertaining to fluorescence microscopy, please refer to the supplementary methods. All cell images were obtained at ×1000 magnification using a ×100 Plan-Fluorite oil immersion objective (Olympus, UK) in combination with low auto-fluorescence immersion oil (Olympus immersion oil type-F). The exposure settings for the lumogallion analyses were fixed at 1 s with fixed light transmission values. Images were obtained using the Cell^D^ software (Olympus, Soft Imaging Solutions, GmbH) package and fluorescent and light channels were overlaid by use of Photoshop (Adobe systems Inc. USA).

### Transmission electron microscopy (TEM)

Grids were stained with 2% *w/v* uranyl acetate as described in the supplementary methods. Samples for TEM were viewed on a JEOL 1230 transmission electron microscope operated at 100.0 kV (spot size 2), equipped with a Megaview III digital camera from Soft Imaging Systems (SIS). Images were obtained on the iTEM universal TEM imaging platform software. Measurements of intracellular ABA particles were made using the Cell^D^ software package and the final editing of electron micrographs for publication was achieved using Photoshop (Adobe systems Inc. USA).

## Results

### Identification of intracellular AlO(OH) particles in THP-1 agar-cell sections

**Figure 1:**
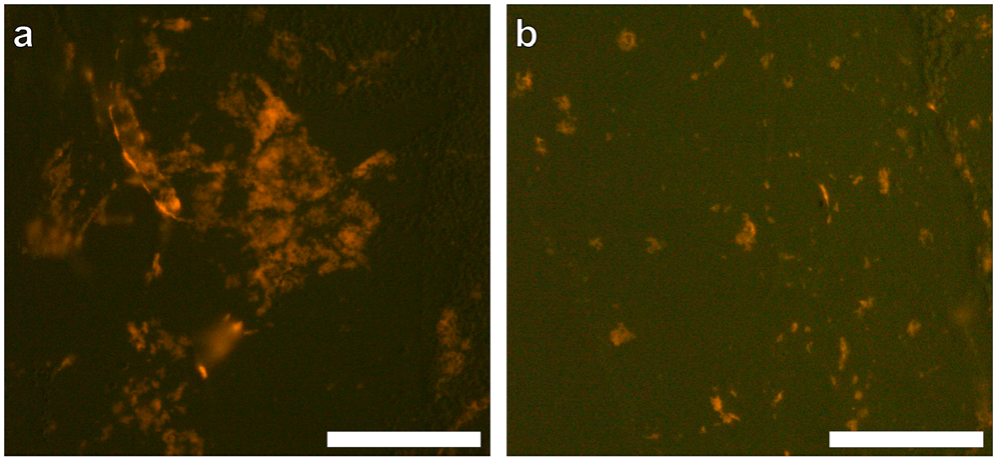
Representative lumogallion fluorescence of agar-paraffin embedded AlO(OH) adjuvants (2 μm sections). AlO(OH) sections were incubated for 24 h in 50 mM PIPES, pH 7.4 containing 100 μM lumogallion. Slides were mounted with Fluoromount™ aqueous mounting medium (Sigma Aldrich). (a). 200 μg/mL AlO(OH)^Brenntag^ adjuvant, (b). 200 μg/mL AlO(OH)^Sigma^ adjuvant. Magnification × 400, scale bars: 50 μm.

Preliminary experiments in which ABA only (AlO(OH)^Sigma^ and AlO(OH)^Brenntag^) were embedded into agar-paraffin and subsequently stained with lumogallion for 24 h (as with THP-1 cell sections) proved successful in positively identifying both ABA as evidenced by an orange fluorescence emission (Fig. 1).

**Figure 2:**
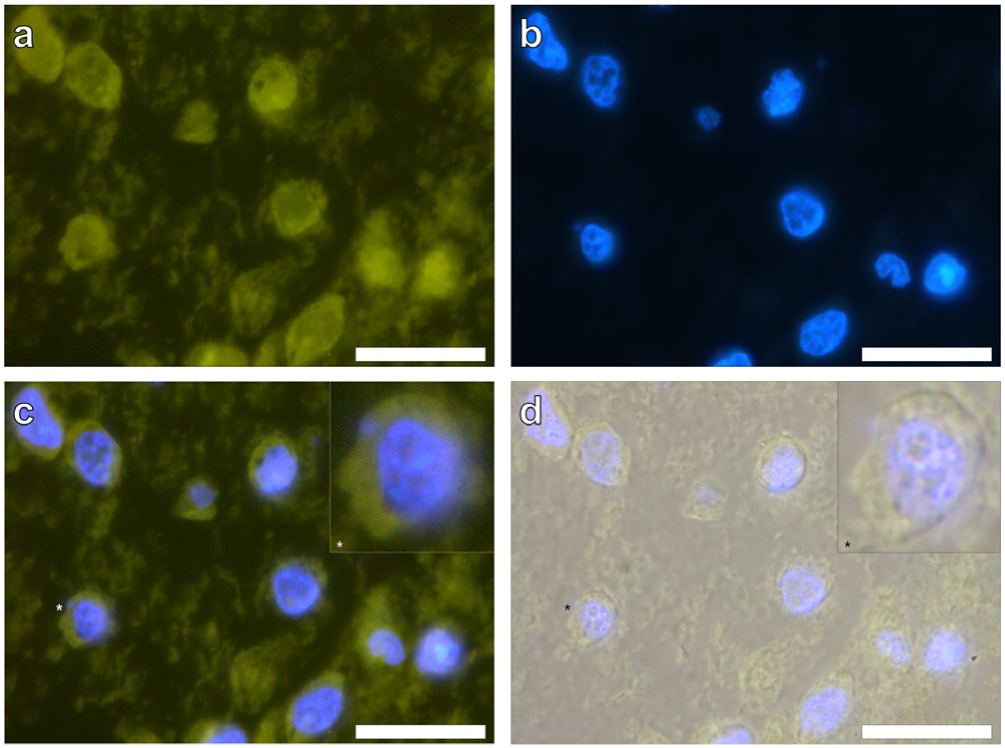
Representative autofluorescence controls of agar-paraffin embedded THP-1 cells (2 μm sections) co-cultured with 200 μg/mL AlO(OH)^Sigma^ adjuvant (24 h). Cell sections were incubated for 24 h in 50 mM PIPES, pH 7.4. Slides were mounted with ProLong® Gold Antifade Reagent with DAPI. Inserts show close-ups of individual cells. (a). autofluorescence (green), (b). DAPI-staining (blue), (c). autofluorescence & DAPI overlay, (d). autofluorescence, DAPI & light overlay. Magnification × 1000, scale bars: 20 μm.

Native THP-1 cells grown in R10 medium in the presence or absence of AlO(OH)^Sigma^ showed green autofluorescence in the absence of the fluor lumogallion (Fig. 2a). Analysis of THP-1 cells containing 200 μg/mL AlO(OH)^Sigma^ revealed that those structures identified produced clear DAPI fluorescence (Fig. 2b–c) confirming the presence of nuclei and hence the positive identification of THP-1 cells. Overlaying of the light channel elucidated that the green autofluorescence observed was most intense within the cell cytoplasm (Fig. 2d).

**Figure 3:**
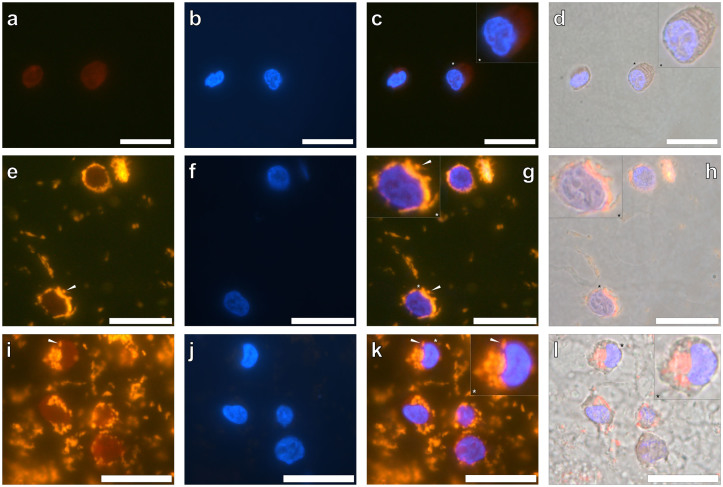
Representative lumogallion staining of agar-paraffin embedded (2 μm sections) native THP-1 cells (a–d), THP-1 cells co-cultured with 50 μg/mL AlO(OH)^Sigma^ adjuvant (24 h) (e–h) & THP-1 cells co-cultured with 200 μg/mL AlO(OH)^Sigma^ adjuvant (24 h) (i–l). Cell sections were incubated for 24 h in 100 μM lumogallion, 50 mM PIPES, pH 7.4. Slides were mounted with ProLong® Gold Antifade Reagent with DAPI. (a)., (e). & (i). lumogallion staining (orange), (b)., (f). & (j). DAPI-staining (blue), (c)., (g). & (k). lumogallion & DAPI overlay, (d)., (h). & (l). lumogallion, DAPI & light overlay. Inserts show close-ups of individual cells and white arrows highlight both individual and distinguishable adjuvant particles. Magnification × 1000, scale bars: 20 μm.

In order to investigate whether AlO(OH)^Sigma^ was taken up by THP-1 cells, the cells were co-cultured in the presence or absence of the ABA, sectioned and then stained with lumogallion. Lumogallion staining of native THP-1 cells revealed a dull orange/brown fluorescence for the cells (Fig. 3a) with DAPI fluorescence confirming the presence of cell nuclei (Fig. 3b & c). Overlaying of the light channel demonstrated that only cells were fluorescing with no detectable fluorescence of the agar-support medium (Fig. 3d).

THP-1 cells co-cultured with 50 μg/mL of the AlO(OH)^Sigma^ adjuvant and stained with lumogallion, produced bright orange fluorescence at their periphery (Fig. 3e) and clear DAPI fluorescence (Fig. 3f). Merging of the fluorescence and light channels revealed that the bright orange fluorescence of the AlO(OH)^Sigma^ adjuvant (Fig. 3g, magnified inset) was contained only within the cytoplasm of THP-1 cells (Fig. 3h). Extracellular ABA was readily identifiable for lumogallion stained THP-1 cells co-cultured with 200 μg/mL AlO(OH)^Sigma^ (Fig. 3i). As with THP-1 cells co-cultured with 50 μg/mL AlO(OH)^Sigma^, spherical particulate-like structures were identified intracellularly in THP-1 cells exhibiting DAPI fluorescence (Fig. 3j & k). The combination of light and fluorescence channels depicted the clear localisation of an orange fluorescence emission within cell cytoplasm and not within cell nuclei (Fig. 3l).

Spherical particulates of AlO(OH)^Sigma^ identified intracellulary in THP-1 cells proved to be consistent in size whether co-cultured with 50 or 200 μg/mL of the ABA. Spherical ABA particulates with an average outer diameter of 0.88 ± 0.17 μm and 0.90 ± 0.14 μm were observed (mean ± SD, *n* = 16) intracellulary in THP-1 cells co-cultured in the presence of 50 and 200 μg/mL of the AlO(OH)^Sigma^ adjuvant, respectively (Table 1).

### Identification of intracellular AlO(OH) particles in cryosectioned THP-1 cells

**Figure 4:**
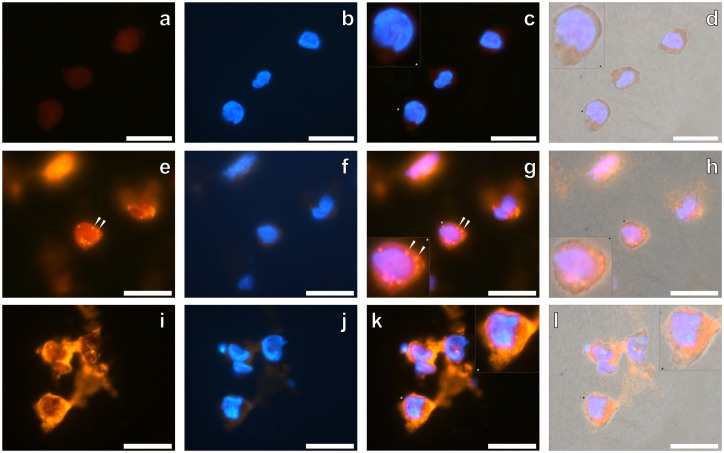
Representative lumogallion staining of cryosectioned (6 μm sections) native THP-1 cells (a–d), THP-1 cells co-cultured with 50 μg/mL AlO(OH)^Brenntag^ adjuvant (24 h) (e–h) & THP-1 cells co-cultured with 200 μg/mL AlO(OH)^Brenntag^ adjuvant (24 h) (i–l). Cell sections were incubated for 24 h in 100 μM lumogallion, 50 mM PIPES, pH 7.4. Slides were mounted with ProLong® Gold Antifade Reagent with DAPI. (a)., (e). & (i). lumogallion staining (orange), (b)., (f). & (j). DAPI-staining (blue), (c)., (g). & (k). lumogallion & DAPI overlay, (d)., (h). & (l). lumogallion, DAPI & light overlay. Inserts show close-ups of individual cells and white arrows highlight both individual and distinguishable adjuvant particles. Magnification × 1000, scale bars: 20 μm.

We next examined whether the clinically approved AlO(OH)^Brenntag^ adjuvant was internalised in THP-1 cells co-cultured in the presence or absence of 50 or 200 μg/mL of the ABA via fluorescence microscopy. Analysis of cryo-sectioned THP-1 cells showed a green autofluorescence of the cells with clear DAPI fluorescence both in the presence or absence of the AlO(OH)^Brenntag^ adjuvant. For lumogallion stained native THP-1 cells cultured in the presence of R10 medium only, a dull orange/brown fluorescence was observed (Fig. 4a). DAPI fluorescence confirmed the presence of cell nuclei (Fig. 4b) which overlaid with the lumogallion fluorescence (Fig. 4c) and light channels confirmed the presence of a dull orange/brown fluorescence within the cytoplasm of THP-1 cells (Fig. 4d).

Cryosectioned THP-1 cells co-cultured in the presence of 50 μg/mL of the AlO(OH)^Brenntag^ adjuvant and stained with lumogallion revealed intracellular particulates of the ABA as identifiable by the observed orange fluorescence emission (Fig. 4e). Overlay of the DAPI fluorescence channel (Fig. 4f) confirmed that THP-1 cells had internalised AlO(OH)^Brenntag^ particulates, predominantly within their cytoplasm with no evidence of ABA being present within cell nuclei (Fig. 4g, magnified inset). Overlay of the light channel confirmed that positive lumogallion staining for ABA was only notable within cell cytoplasm with little trace of extracellular ABA (Fig. 4h).

Similar observations were noted for THP-1 cells co-cultured in the presence of 200 μg/mL AlO(OH)^Brenntag^, with a strong orange fluorescence emission being observed at the periphery (Fig. 4i) of positively identified THP-1 cells (Fig. 4j & k). Overlay of the light with the lumogallion and DAPI fluorescence channels confirmed the presence of a strong orange fluorescence emission within the cell cytoplasm (Fig. 4l, magnified inset). Identification of individual particulate-like structures was sometimes hindered due to the intense orange fluorescence observed. Measurements of intracellular AlO(OH)^Brenntag^ particulates within THP-1 cells was however possible with cells co-cultured in 50 or 200 μg/mL of the ABA giving average outer diameters of 1.09 ± 0.23 μm and 1.00 ± 0.10 μm (mean ± SD, *n* = 16), respectively. The dimensions of intracellular AlO(OH)^Brenntag^-based particulates was therefore in close agreement to THP-1 cells co-cultured in the presence of the AlO(OH)^Sigma^ adjuvant (Table 1).

### The complementary use of transmission electron microscopy confirmed the presence of intracellular particulate AlO(OH) adjuvant within THP-1 cells

**Figure 5:**
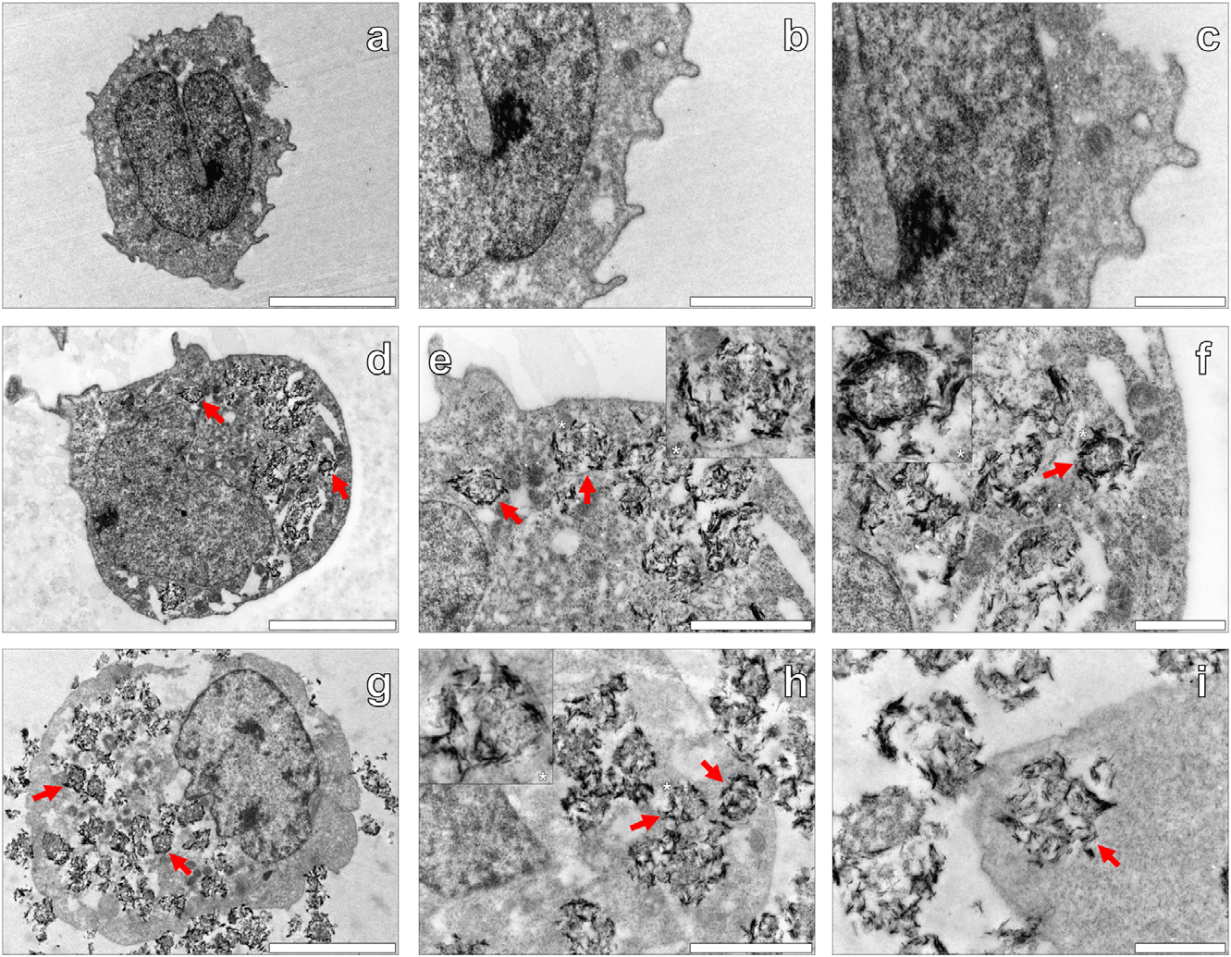
Representative electron micrographs from TEM of Spurr resin-sectioned (100 nm sections) native THP-1 cells (a–c), THP-1 cells co-cultured with 50 μg/mL AlO(OH)^Sigma^ adjuvant (24 h) (d–f) & THP-1 cells co-cultured with 200 μg/mL AlO(OH)^Sigma^ adjuvant (24 h) (g–i). Cell resin-sections were stained for 20 min with 2% ethanolic uranyl acetate, rinsed with 30% ethanol followed by ultrapure water and finally allowed 24 h drying time prior to analysis via TEM. Inserts show close-ups of intracellular adjuvant particles contained within vesicle-like structures and the red arrows highlight their presence within the respective cell images. Magnification & scale bars: (a)., (d). & (g). × 8 K, 5 μm, (b)., (e). & (h). × 20 K, 2 μm, (c)., (f). & (i). × 30 K, 1 μm, respectively.

To support any observations of intracellular AlO(OH) particulates within THP-1 cells, cells co-cultured in the presence or absence of the AlO(OH)^Sigma^ adjuvant were embedded into resin and analysed by transmission electron microscopy (TEM). TEM of native THP-1 cells cultured in R10 medium only allowed for the detection of cells (Fig. 5a) with clear distinction between cell cytoplasm and nuclei (Fig. 5a–c). Native THP-1 cells were found to have a dense granular cytoplasm with the entire cell free of any particulate material (Fig. 5c).

For those cells co-cultured in the presence of 50 μg/mL of the AlO(OH)^Sigma^ adjuvant, electron dense particulates were observed within the cytoplasm of THP-1 cells (Fig. 5d). Higher magnifications revealed the presence of needle-like particulates (Fig. 5e & f) in aggregates with an average size of 1.04 ± 0.35 μm (mean ± SD, *n* = 16) (Table 1). Those particulates observed were found to be present in the cell cytoplasm only and contained within vesicle-like structures.

THP-1 cells co-cultured with 200 μg/mL of the AlO(OH)^Sigma^ adjuvant revealed aggregated particulates of the ABA solely present within the cell cytoplasm (Fig. 5g). Extracellular ABA was also found within the agar support medium as with the lumogallion fluorescence analyses of the cells (Fig. 3i & Fig. 4i). Higher magnifications revealed that internalised AlO(OH) particulates were found in vesicle-like structures (Fig. 5h & i) as with cells co-cultured in lower concentrations of the ABA with an average size of 1.20 ± 0.26 μm (mean ± SD, *n* = 16) (Table 1). It was also noted that the cell cytoplasm of THP-1 cells co-cultured in the presence of the ABA (Fig. 5d & Fig. 5g) exhibited a loss of integrity in comparison to native cells (Fig. 5a).

### Confocal microscopy confirms the presence of intracellular AlO(OH) in non-sectioned THP-1 cells

To ascertain whether AlO(OH)^Brenntag^ was internalised within THP-1 cells whilst avoiding sectioning and the potential loss or disturbance of intracellular particulates, cells cultured in the absence or presence of the ABA were stained with lumogallion and analysed by confocal microscopy. Cells were counter-stained with DAPI for the identification of cell nuclei. Merging of both DAPI and lumogallion fluorescence channels for THP-1 cells co-cultured with 50 μg/mL AlO(OH)^Brenntag^ showed positive lumogallion staining for aluminium observed as spherical particulates contained within the cytoplasm of cells with a lack of particulate material localised in cell nuclei (see Supplementary Fig. 1a). Merging of the two channels for native THP-1 cells revealed positive DAPI staining only, confirming the presence of cell nuclei (see Supplementary Fig. 1b).

## Discussion

We have investigated whether a clinically approved AlO(OH)^Brenntag^ adjuvant and an experimental AlO(OH)^Sigma^ adjuvant of similar formulation can enter a monocytic THP-1 cell line. Whilst studies have pertained to the presence of ABA inside APCs and cells of monocytic origin, none have shown the unequivocal presence of intracellular ABA^6^,^12^,^15^,^21^,^29^. A recent study has demonstrated the presence of *in vivo* injected aluminium adjuvant in rat at a muscular as opposed to a cellular level using lumogallion^30^. The direct identification of intracellular particulates of AlO(OH)-based adjuvants were therefore sought via the use of lumogallion as a sensitive molecular probe for aluminium^24^,^25^. The use of lumogallion also ensured that aluminium was detected inside cells as opposed to the identification of molecular markers which in themselves may be internalised with antigen in the absence of the attached ABA, or may have an influence upon their cellular uptake.

It was not known whether lumogallion could bind to particulate ABA to provide a characteristic fluorescence emission at *ca* 590 nm (orange)^28^. Whilst easily distinguishable from the agar support medium, those AlO(OH) particulates observed were found to fluoresce at low intensity (Fig. 1). As AlO(OH) possesses a crystalline structure with a degree of symmetry^31^, a high proportion of available aluminium sites (Al^3+^) will be held internally forming crystal contacts, thereby occluding their binding to lumogallion with only external sites able to bind. In spite of these observations however both the clinical and experimental AlO(OH) formulations could be detected. To our knowledge this is the first report of the use of lumogallion for the unequivocal detection of intracellular ABA.

For the investigation of the potential intracellular uptake of AlO(OH), a cultured human THP-1 cell line of monocytic origin was used in order to investigate a relevant *in vitro* model used in separate reports of the effects of ABA on mitochondrial activity and phagosomal pathways of phagocytosing cells^32^. Morin has previously been used to detect internalised ABA conjugated to antigens^11^ via a green fluorescence emission at *ca* 510 nm^18^. Therefore the establishment of autofluorescence of any given cell line is critical in order to rule out the possibility of the green fluorescence signal observed being attributable to the cellular components rather than that of internalised ABA.

For the analysis of cell sections under the lumogallion fluorescence channel, a long pass emission filter was used allowing emitted wavelengths of or greater than 510 nm to pass through hence explaining the observed green autofluorescence for cells (Fig. 2). Due to the aforementioned sensitivity issues of morin binding to divalent metal ions to produce false positives, lumogallion provides an attractive approach to the identification of ABA with high sensitivity at a physiologically relevant pH.

A uniform orange fluorescence was observed for cells co-cultured in the presence of AlO(OH) in regions of the cells found to be devoid of particulate AlO(OH). The fluorescence observed was found to be less intense than that of positively identified particulates (e.g. Fig. 4e) but more intense than the dull orange/brown fluorescence observed for native cells (e.g. Fig. 4a), likely explained by the use of a long pass emission filter. Therefore we cannot rule out the possibility of soluble Al^3+^ being present within cell cytoplasm and/or nuclei, as inferred by the lumogallion analyses. Particulates for both the clinically approved and experimental AlO(OH) formulations were localised within the cell cytoplasm of THP-1 cells. These observations were supported via TEM in which particulate AlO(OH)^Sigma^ was localised within vesicle-like structures in the cytoplasm of THP-1 cells only (Fig. 5), in similar size aggregates of *ca* 1 μm (Table 1).

Fluorescence microscopy and TEM both required cells to be sectioned to allow for the exposure of intracellular AlO(OH) to lumogallion and uranyl acetate, respectively. To rule out the possibility of particulate AlO(OH) being observed in THP-1 cells as a result of artefacts arising from the sectioning and embedding procedures^33^, confocal microscopy of whole cells was performed. Confocal microscopy of living cells stained with lumogallion demonstrated the presence of particulate AlO(OH) within the cell cytoplasm only thereby confirming that ABA were deposited intracellularly and did not arise from extracellular particulates being deposited on the surface of newly exposed cell sections (see Supplementary Fig. 1).

Research investigating the phagosomal pathways of THP-1 cells in the presence of AlO(OH) implicated that phagocytosis of particulate ABA results in the entrapment of particulate ABA in phagosomes^32^. It was concluded that the subsequent maturation of phagosomes into phagolysosomes resulted in the acidification of their interior causing the pH to fall to *ca* 4.5 to 5. Under these acidic conditions it is likely that the solubility of the internalised ABA will be increased^32^ resulting in the greater availability of intracellular Al^3+^. Interestingly, for the unequivocal identification of intracellular AlO(OH) demonstrated herein, a more intense orange fluorescence emission was observed for intracellular versus extracellular ABA particulates (e.g., Fig. 3e & 4i). As lumogallion is known to bind 1:1 with Al^3+^ a greater availability of the metal ion would result in increased complex formation, thereby explaining the intense fluorescence observed for intracellular AlO(OH). Our results therefore support the phagocytosis of ABAs by monocytic cells.

A study investigating the uptake of ovalbumin adsorbed onto the surface of alpha aluminium oxide nanoparticles (α-Al_2_O_3_-OVA) by DCs showed electron dense particles internalised within autophagosomes and autlolysosomes by TEM^21^. Those aggregates observed were found to be *ca* 1 μm in size^21^ and therefore in the same size range for intracellular AlO(OH) shown herein. These results taken together suggest that the intracellular uptake of ABA by cells of monocytic origin, occurs via phagocytotic mechanisms^21^,^32^. Within this research we have shown that lumogallion provides an excellent molecular probe for ABA, allowing for the fate of ABA to be determined in a relevant *in vitro* cell line^32^.

The adsorption of inherent antigen in the form of serum proteins including bovine serum albumin included in the 10% foetal calf serum used for cell culture, may have affected the uptake of the AlO(OH) adjuvants used within this study. Further studies are currently underway to address the influences of co-adsorbed antigens on the cellular uptake of aluminium adjuvants. In addition we now aim to ascertain whether internalised aluminium adjuvant has antigen associated with it. The unequivocal identification of intracellular ABA may prove essential for the understanding of the adjuvanticity of ABAs administered in human vaccinations.

## Author Contributions

M.M., C.E., H.E., P.S. and E.S. designed the study. M.M. and C.E. wrote the manuscript. M.M. performed all fluorescence microscopy and TEM analyses of THP-1 cells and prepared the figures. H.E. prepared all THP-1 cell treatments for microscopy and performed all confocal microscopy. A.D. and P.S. performed the cryosectioning of THP-1 cells. All authors read and commented upon the manuscript.

## Supplementary Material

Supplementary Information Supplementary information

## Figures and Tables

**Table 1:** Intracellular AlO(OH) particle sizes (outer diameter, OD) in THP-1 cells co-cultured with 50 or 200 μg/mL of either AlO(OH)^Sigma^ (1) or AlO(OH)^Brenntag^ (2) adjuvant for 24 h. Cells were sectioned via three different methods: agar-paraffin sectioning, cryosectioning or transmission electron microscopy (TEM). Visualisation of intracellular adjuvant particles for agar-paraffin and cryosectioned THP-1 cells was achieved by fluorescent staining with lumogallion and for TEM by staining with 2% uranyl acetate. Particle sizes were determined by use of the Cell^D^ software suite (Olympus) and mean particle size ± SD are shown (*n* = 16)

	Intracellular AlO(OH) particle size (OD)/μm		
[AlO(OH)]/μg/mL	^(1)^THP-1 Agar-paraffin^Sigma^	^(2)^THP-1 Cryo^Brenntag^	^(1)^THP-1 TEM^Sigma^
50	0.88 ± 0.17	1.09 ± 0.23	1.04 ± 0.35
200	0.90 ± 0.14	1.00 ± 0.10	1.20 ± 0.26
