# Supplementary material for: Unequivocal identification of intracellular aluminium adjuvant in a monocytic THP-1 cell line
Source: Sci Rep. 2014 Sep 5;4:6287. doi: 10.1038/srep06287 (PMC4155332; doi:10.1038/srep06287)
Supplement: Supplementary Information [file srep06287-s1.pdf]

Supplementary information

Title:

Unequivocal identification of intracellular aluminium adjuvant in a monocytic THP-1 cell line.

Author List:

Dr Matthew J. Mold: m.j.mold@keele.ac.uk

Prof Håkan L. E. Eriksson: hakan.eriksson@mah.se

Dr Anna Darabi (Assistant Prof): anna.darabi@med.lu.se

Prof Peter Siesjö: peter.siesjo@med.lu.se

Emma M. Shardlow: e.shardlow@keele.ac.uk

Prof Christopher Exley*: c.exley@keele.ac.uk

*****_Corresponding author_

Postal addresses:

[a]The Birchall Centre, Lennard-Jones Laboratories, Keele University, Keele, Staffordshire, ST5 5BG, UK; [b]Department of Biomedical Laboratory Science, Faculty of Health and Society, Malmö University, SE-205 06 Malmö, Sweden; [c]Glioma Immunotherapy Group, Department of Clinical Sciences, BMC D14,Lund University, SE-221 84 Lund, Sweden.

Supplementary Figure

Confocal microscopy of THP-1 cells co-cultured in R10 medium in the absence or presence of $AlO(OH)^{Brenntag}$

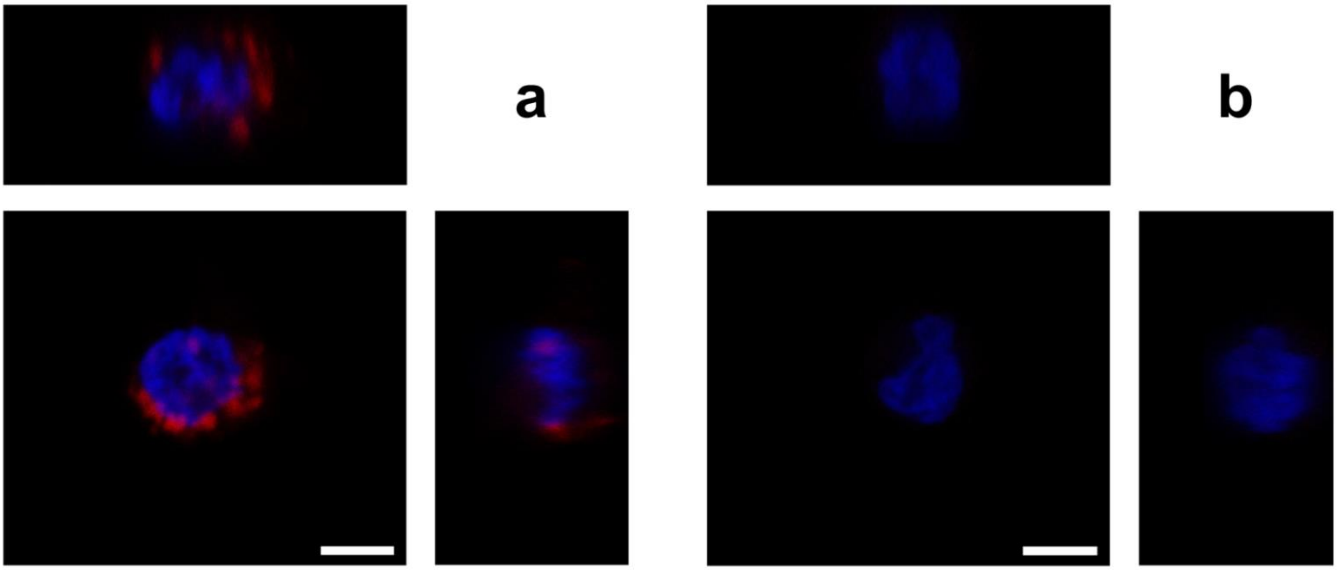

Supplementary Figure 1 Representative confocal imaging of non-sectioned THP-1 cells cultured in R10 medium in the presence (**a**) or absence (**b**) of 50µg/mL $AlO(OH)^{Brenntag}$ adjuvant and stained in culture with lumogallion. **a**. Confocal images of a z series containing 59 slices of cells co-cultured with Al adjuvant and stained with lumogallion. Lower left micrograph shows the xy projection of the middle of the z stack, upper micrograph xz projections and right micrograph yz projections. **b**. Confocal images of a z series containing 45 slices of cells cultured in R10 medium and stained with lumogallion. Projections are as with **a**. Merged images depict both lumogallion (orange) and DAPI (blue) fluorescence. All scale bars: 5µm.

Supplementary Methods

Pre-embedding THP-1 cells into an agar support medium

THP-1 cells were initially pelleted via centrifugation for 8 min at 5000 x g (these centrifuge settings were used throughout). The supernatant was then removed (70% ethanol) and cells were re-suspended in a PIPES-based buffer (150mM NaCl, 50mM PIPES, pH 7.4) and centrifuged once more to re-pellet the cells. Each respective THP-1 cell treatment was washed twice more using the same method. The cell treatments were finally re-suspended in the same PIPES-based buffer and transferred into a BEEM® capsule (Agar Scientific, UK) to a final volume of 20µL. Approximately 300,000 cells for each of the treatments were isolated for embedding from THP-1 cell stocks, as calculated from the respective cell densities from flow cytometry.

For pre-embedding an initial agar stock solution of 5% *w/v* was prepared which was subsequently heated to between 95 – 100°C by use of a water bath. Once the agar was fully dissolved (as evidenced by a clear solution), 30µL was transferred directly into each BEEM® capsule by use of heated pipette tips. Each capsule was immediately capped, vortexed briefly and finally centrifuged for approximately 10 s. The final pelleted 3% *w/v* agar 50µL cell blocks were allowed a minimum of 1 h to set at 4°C, prior to dehydration procedures.

Dehydration and clearing

Processing of the agar-cell blocks was staggered to allow for accurate incubation timings throughout the embedding process. Agar-cell blocks were transferred through a graded ethanol series from 30 – 100 % *v/v* ethanol (HPLC grade used throughout) with 20 min allowed in each ethanol concentration (30, 50, 70, 90, 97 and (2x) 100 % *v/v* ethanol) to ensure gradual and sufficient dehydration of the blocks. Following dehydration agar-cell blocks for paraffin embedding were cleared by transferring the blocks into 1.0mL of Histo-Clear, a d-Limonene based reagent (National Diagnostics, USA) for 20 min, with one change of fresh Histo-Clear half way through.

Infiltration and embedding of agar-cell blocks

Cleared agar-cell blocks were infiltrated by placing the blocks into melted paraffin at 60°C in stainless steel histology embedding moulds for 35 – 40 min. The temperature of the paraffin wax was carefully monitored throughout and never exceeded 65°C. Once infiltrated, the agar-cell blocks were transferred into smaller stainless steel histology embedding moulds filled with melted paraffin wax and orientated as necessary. An embedding cassette was placed over the mould and filled with melted paraffin wax and the whole assembly was transferred onto ice for approximately 15 min to cool the wax rapidly, thereby minimising imperfections in the final block. The embedded blocks were set fully at 4°C overnight prior to sectioning.

Resin embedding for transmission electron microscopy

THP-1 cells co-cultured in complete R10 medium in the presence or absence of 50 or 200μg/mL $AlO(OH)^{Sigma}$ adjuvant (24 h), were pre-embedded into agar and gradually dehydrated to 70% *v/v* ethanol and then stored at $4^{o}C$ overnight. THP-1 agar-cell blocks were dehydrated with 100% ethanol (as with those blocks prepared for paraffin embedding) and were further dehydrated prior to resin-embedding by transfer into dry (100%) ethanol (containing molecular sieve) for 20 min.

Fresh Spurr resin was prepared by sequentially weighing 13g NSA (nonenyl succinic anhydride, R1054), 5g ERL 4221 (3,4-epoxycyclohexylmethyl 3,4-epoxycyclohexyl carboxylate), 3g DER (diglycidyl ether of polypropylene glycol, R1074) and 0.2g S-1 (DMAE, dimethylaminoethanol, R1067) (Agar Scientific, UK) into a disposable polyethylene cup. Once all the components were added they were subsequently mixed slowly by use of a variable speed electric stirrer for a maximum of 15 min. Infiltration of agar-cell blocks was achieved gradually by first transferring the blocks into 3:1 dry ethanol : Spurr resin for 2 h, 1:1 dry 100% ethanol : Spurr resin for 2 h and finally 1:3 dry 100% ethanol overnight, all at ambient temperature. Full infiltration of agar-cell blocks was achieved by performing three changes of fresh Spurr resin over 8 h after which blocks were arranged into moulds and polymerised for a minimum of 16 h in a resin oven at $60^{o}C$.

Microtomy

Paraffin-embedded agar-cell blocks were sectioned by use of a Leica RM2025 rotary microtome fitted with Leica 819 low profile microtomy blades (Leica Biosystems, GmbH,

Germany). A section thickness of 2μm was found to be optimal and was therefore used throughout. Paraffin sections were floated out on ultrapure water at 50°C by use of paraffin floatation bath for 30 s. Sections were then caught on glass slides and initially dried at 35°C in a slide heater. The sections were dried fully overnight at ambient temperature prior to de-waxing and staining procedures.

Resin-embedded agar-cell blocks were sectioned by use of a Leica ultracut UCT ultramicrotome fitted with glass blades (Leica Biosystems, GmbH, Germany). A plastic trough was attached to the blade allowing for 100nm sections to be floated out on ultrapure water and caught on non-coated 200 mesh, thin bar, 3.05mm copper grids (Athene, UK). Grids were allowed a minimum of 24 h drying time, prior to staining.

Fluorescence microscopy

DAPI-mounted THP-1 cell sections were viewed using an Olympus BX50 fluorescence microscope with a BX-FLA reflected light fluorescence attachment, equipped with a mercury burner and a vertical illuminator. For DAPI imaging, a U-MWU2 fluorescence filter cube was used (bandpass excitation filter: 300 – 385nm, dichromatic mirror: 400nm, longpass emission filter: 420nm) and for lumogallion imaging a U-MNIB3 fluorescence filter cube was used (bandpass excitation filter: 470 – 495nm, dichromatic mirror: 505nm, longpass emission filter: 510nm) (both from Olympus, UK).

Transmission electron microscopy (TEM)

For TEM, 2% *w/v* uranyl acetate was prepared fresh in 70% *v/v* ethanol in capped glass vials and then placed in an ultrasonic bath for a maximum of 10 min. The stain was then centrifuged at high speed for 10 min prior to use. 100nm resin agar-cell sections were negatively stained for TEM by insertion into the uranyl acetate stain for 20 min. Following staining with uranyl acetate, grids were removed, wicked and dipped into a large volume of 30% ethanol 20 times to remove any excess uranyl acetate. Grids were then re-wicked, rinsed twice more by dipping the grids 10 times respectively in fresh ultrapure water and were wicked in between rinses. Grids were finally re-wicked following this step, covered and allowed to dry for up to 24 h, prior to analysis via TEM.

Confocal Microscopy

THP-1 cells, 0.25 x 10^6 cells/ml, were growth in the presence or absence of 50µg/mL $AlO(OH)^{Brenntag}$ adjuvant in R10 medium. After 16 h lumogallion was added to a final concentration 10 µM. The culture was continued for another 16 h before the cells were harvested by centrifugation at 900 x g during 5 min. Collected cells were fixed by re-suspension in 50µl 1 % PFA and mounted on slides using ProLong® Gold Antifade Reagent (Molecular Probes). Images were obtained using a LSM 780 laser scanning microscope (Carl Zeiss AG, Germany) with ZEN software. Lumogallion fluorescence was detected using the 488 nm laser and Alexa 546 as emission setting.
